# Endoscopic ultrasound‐guided hepaticogastrostomy using a partially covered metal stent in patients with malignant biliary obstruction after failed Endoscopic retrograde cholangiopancreatography

**DOI:** 10.1002/jgh3.12386

**Published:** 2020-07-07

**Authors:** James Emmanuel, Haniza Omar, Lee T See

**Affiliations:** ^1^ Department of Gastroenterology and Hepatology Selayang Hospital Selangor Malaysia

**Keywords:** endoscopic ultrasound‐guided biliary drainage, malignant biliary obstruction, partially covered metal stent

## Abstract

**Background and Aim:**

The advent of endoscopic ultrasound‐guided biliary drainage (EUS‐BD) has provided an inimitable alternative for gaining biliary access in patients who fail conventional endoscopic drainage. The antimigratory features of the partially covered metal stent (PCMS), namely, the flange head and uncovered portion of the stent, makes it a valuable option in patients undergoing EUS‐guided hepaticogastrostomy (EUS‐HGS). The aim of the study is to evaluate the clinical outcome of EUS‐BD via the hepaticogastrostomy approach using PCMS in patients with malignant biliary obstruction after failed ERCP.

**Methods:**

This is a single‐center retrospective observational study of patients with malignant biliary obstruction undergoing EUS‐HGS after failed ERCP between January 2018 and May 2019. The end‐point of the study was to assess the technical and clinical success rate, as well as the stent‐ and procedure‐related complications.

**Results:**

There were 20 subjects in this study. The average age was 71.8 ± 7.6 years. Most patients were male, 16 (80%). Inaccessible papillae was the most common indication for this procedure, 16 (80%). Technical success was achieved in all patients. The average procedural time was 39.9 ± 1.3 min. Mean preprocedural bilirubin levels were 348.6 ± 28.8 and subsequently decreased to 108.94 ± 37.1 μmol/L at 2 weeks postprocedure. The clinical success rate was 95% (19/20), with one patient requiring percutaneous transhepatic biliary drainage (PTBD). There were no stent‐ or procedure‐related complications reported in this study.

**Conclusion:**

EUS‐HGS with PCMS is a feasible, effective, and safe alternative for biliary decompression in patients with failed endoscopic retrograde cholangiopancreatography (ERCP).

## Graphical abstract

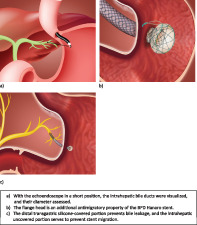

The partially covered metal stent (PCMS) with the covered distal transgastric portion and the proximal uncovered portion within the intrahepatic ducts.

## Introduction

The advent of endoscopic ultrasound‐guided biliary drainage (EUS‐BD) has provided an inimitable alternative for biliary access in patients who fail conventional endoscopic drainage.^1^ This procedure has amalgamated technical expertise from both EUS and endoscopic retrograde cholangiopancreaticography (ERCP) to achieve biliary drainage when the former is unsuccessful.^2^, ^3^ Failure in ERCP is encountered in 5–10% of the cases due to surgically altered anatomy, gastric outlet obstruction, periampullary diverticulum, and complete ductal obstructions.^1^, ^4^, ^5^ Alternatives after an unsuccessful ERCP include performing a PTBD or pursuing a surgical intervention. Both options, however, have been fraught with multiple potential adverse events. In PTBD, both procedure‐ and drainage‐related complications rates have been reported of up to 32%.^4^, ^6^, ^7^ Surgical intervention, on the other hand, is associated with substantial morbidity (35–50%) and mortality (10–15%).^8^, ^9^, ^10^

EUS‐BD represents a less‐invasive procedure to the aforementioned options. There is no consensus on the technique of choice in EUS‐BD, although among some endoscopists, choledochoduodenostomy (CDS) is the preferred option due to its lower complication rates.^1^, ^11^, ^12^ Despite its technical intricacies, the EUS‐guided hepaticogastrostomy (EUS‐HGS) procedure possesses the added advantage of having the most extensive indications, primarily papilla inaccessibility, and surgically altered anatomy.^8^, ^13^

The first EUS‐HGS with a plastic stent was reported by Burmester *et al*.^14^ Over the years, the application of plastic stents in EUS‐HGS have evolved to the utilization of metal stents due to its longer patency and its ability to exert tamponade in the event of bleeding from the gastric wall.^11^, ^13^ The conception of the partially covered metal stent (PCMS) has further addressed the shortcomings encountered with previous stents owing to its hybrid nature. The distal transgastric silicone‐covered portion prevents bile leakage, and the intrahepatic uncovered portion serves to prevent stent migration.^8^, ^9^, ^15^ The flange head is an additional antimigratory property of the BPD Hanaro stent, and in the event of migration, the proximal lasso permits repositioning as a final failsafe mechanism.^15^ The purpose of this study was to evaluate the clinical outcome of EUS‐HGS using a PCMS in patients with malignant biliary obstruction after failed ERCP.

## Methods

This is a single‐center retrospective case review of patients with malignant biliary obstruction undergoing EUS‐HGS between January 2018 and May 2019 after failed ERCP. The procedure was performed by a single endoscopist who averages approximately 1900 EUS procedures and 1100 ERCP procedures a year. Written informed consent was obtained from all patients. Demographic and clinical characteristics, as well as procedure‐ and stent‐related complications, of all the patients who agreed to undergo EUS‐HGS were retrospectively evaluated. The data were retrieved from the electronic medical records and classified based on published literature to facilitate analysis. Technical success was defined as the success in deploying the PCMS stent along with the flow of contrast medium and/or bile through the stent. Clinical success was defined as a reduction in serum bilirubin level of 50% or more within 2 weeks following the HGS procedure. Complications were defined as any procedure‐ or stent‐related complication, including stent migration, stent obstruction, bile leakage with or without bile peritonitis, cholangitis, and pneumoperitoneum. Descriptive statistics were used to summarize the baseline characteristics of patients. Continuous variables were expressed as the mean ± SD, whereas qualitative variables were expressed as frequencies and percentages. *P* < 0.05 was considered statistically significant.

### *Patients*

We conducted a retrospective study on patients who underwent EUS‐HGS using a BPD Hanaro PCMS in our center between 1st January 2018 and 31st May 2019. There were 20 patients included in this study. Inclusion criteria were patients with unresectable malignant biliary obstruction and clinical jaundice who had failed biliary decompression via ERCP. Exclusion criteria were patients who had tumor infiltration along the stomach wall and those that lacked left intrahepatic duct dilatation (<5 mm).

### *Device*

All patients underwent the procedure using the BPD Hanaro PCMS, which had a delivery device diameter of 8.5 fr. The stent diameter was 10 mm, with a flange diameter of 20 mm. The stent used was either 8 or 10 cm in length. There was a fixed 3‐cm uncovered portion that was identical for both stents. The additional features of this stent included 12 radiopaque markers on both ends of the stent for precise stent placement.

### *Hepaticogastrostomy procedure*

All procedures were performed under conscious sedation using a Fujinon EG 580UT linear echoendoscope with a 3.8‐mm working channel. The procedures were carried out with the patient in supine position under sedation using a combination of intravenous midazolam and pethidine. Prophylactic IV ciprofloxacin was administered prior to the procedure. Endoscopic observation was carried out to exclude tumor infiltration of the stomach wall, which would preclude performing this procedure. With the echoendoscope in a short position, the intrahepatic bile ducts were visualized, and their diameter assessed. After identifying neighboring vasculature using the Color Doppler, a 19‐gauge needle was used to gain access to a dilated peripheral branch of the left intrahepatic system in Segment 3 (B3). Bile was aspirated to confirm the position of the needle, and this was followed by contrast instillation to visualize the biliary system under fluoroscopy. With the aid of a 6 Fr cystotome, the diameter of the tract was increased. A partially covered Self‐Expandable Metal Stent (SEMS)with a proximal covered portion (BPD Hanaro Stent; M.I. Tech, Korea) was then introduced to create a communication between the left intrahepatic duct and the gastric lumen.

## Results

Twenty patients were included in this study.

### *Patients baseline characteristics*

The baseline characteristics of the patients are summarized in Table 1. The average age of the patients was 71.8 ± 7.6 years. We had a male preponderance of 16 (80%) male patients and 4 female patients. Pancreatic cancer was the most common underlying primary disease, followed by periampullary tumor, four (20%); cholangiocarcinoma, two (10%); and metastatic colon cancer, one (5%).

**TABLE 1:** Patients’ baseline characteristics

Parameters	Value
Total number of patients	20
Age (years), mean ± SD	71.8 ± 7.60
Gender (male/female), *n* (%)	16 (80%)/4 (20%)
Primary disease	
Pancreatic cancer, *n* (%)	13 (65%)
Periampullary tumor, *n* (%)	4 (20%)
Cholangiocarcinoma, *n* (%)	2 (10%)
Metastatic colon cancer, *n* (%)	1 (5%)

### *Indications of the procedure*

**Figure 1:**
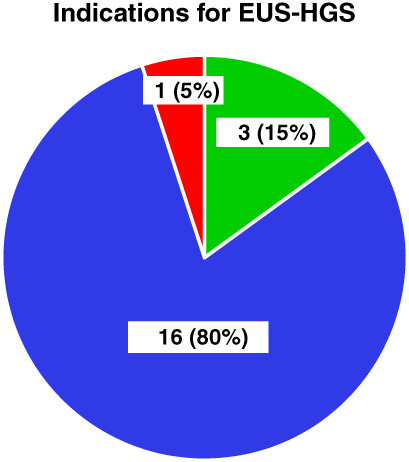
Indications for endoscopic ultrasound‐guided hepaticogastrostomy. 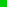, failed/incomplete cannulation; 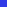, inaccessible papillae; 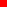, surgically altered anatomy.

The indications for the use of the EUS‐HGS procedure in our cohort of patients were failure to access the papilla secondary to malignant involvement, occluding tumor preventing standard biliary cannulation, and altered anatomy by previous surgery. The indications for the procedure are summarized in Figure 1. Inaccessible papillae were the most common indication for this procedure, 16 (80%), and a subset of these patient either had underlying Type 1 or 2 bilioduodenal stenosis. The least common indication for the procedure was due to surgically altered anatomy.

### *HGS* *procedure*

The details of the HGS procedure are summarized in Table 2.

**Table 2:** Details of the hepaticogastrostomy procedure

No	Age	Primary disease	Pattern/location of obstruction	Reason for ERCP failure	Ascitis	Technical success	Clinical success	Stent length (cm)
1.	81	Ca head of Pancreas	Distal CBD	Inaccessible papillae	No	Yes	Yes	10
2.	73	Ca head of Pancreas	Distal CBD	Inaccessible papillae	No	Yes	Yes	10
3.	78	Ca Colon with duodenal mass	Distal CBD	Surgically altered anatomy	No	Yes	No	10
4.	67	Cholangiocarcinoma	Distal CBD	Failed/incomplete cannulation	No	Yes	Yes	10
5.	77	Periampullary tumor	Distal CBD	Inaccessible papillae	Yes	Yes	Yes	10
6.	82	Periampullary tumor	Distal CBD	Inaccessible papillae	Yes	Yes	Yes	10
7.	60	Ca head of Pancreas	Distal CBD	Inaccessible papillae	No	Yes	Yes	10
8.	81	Ca head of Pancreas	Distal CBD	Inaccessible papillae	No	Yes	Yes	10
9.	63	Ca head of Pancreas	Distal CBD	Inaccessible papillae	No	Yes	Yes	8
10.	78	Periampullary tumor	Distal CBD	Inaccessible papillae	No	Yes	Yes	8
11.	71	Ca head of Pancreas	Distal CBD	Inaccessible papillae	No	Yes	Yes	8
12.	63	Ca head of Pancreas	Distal CBD	Failed/incomplete cannulation	Yes	Yes	Yes	10
13.	84	Ca head of Pancreas	Distal CBD	Inaccessible papillae	No	Yes	Yes	8
14.	68	Ca head of Pancreas	Distal CBD	Inaccessible papillae	No	Yes	Yes	8
15.	60	Ca head of Pancreas	Distal CBD	Inaccessible papillae	No	Yes	Yes	8
16.	74	Cholangiocarcinoma	Proximal CBD	Failed/incomplete cannulation	Yes	Yes	Yes	10
17.	64	Periampullary tumor	Distal CBD	Inaccessible papillae	Yes	Yes	Yes	10
18.	70	Ca head of Pancreas	Distal CBD	Inaccessible papillae	No	Yes	Yes	10
19.	76	Ca head of Pancreas	Distal CBD	Inaccessible papillae	No	Yes	Yes	10
20.	67	Ca head of Pancreas	Distal CBD	Inaccessible papillae	No	Yes	Yes	8

### *Procedural outcome*

**Figure 2:**
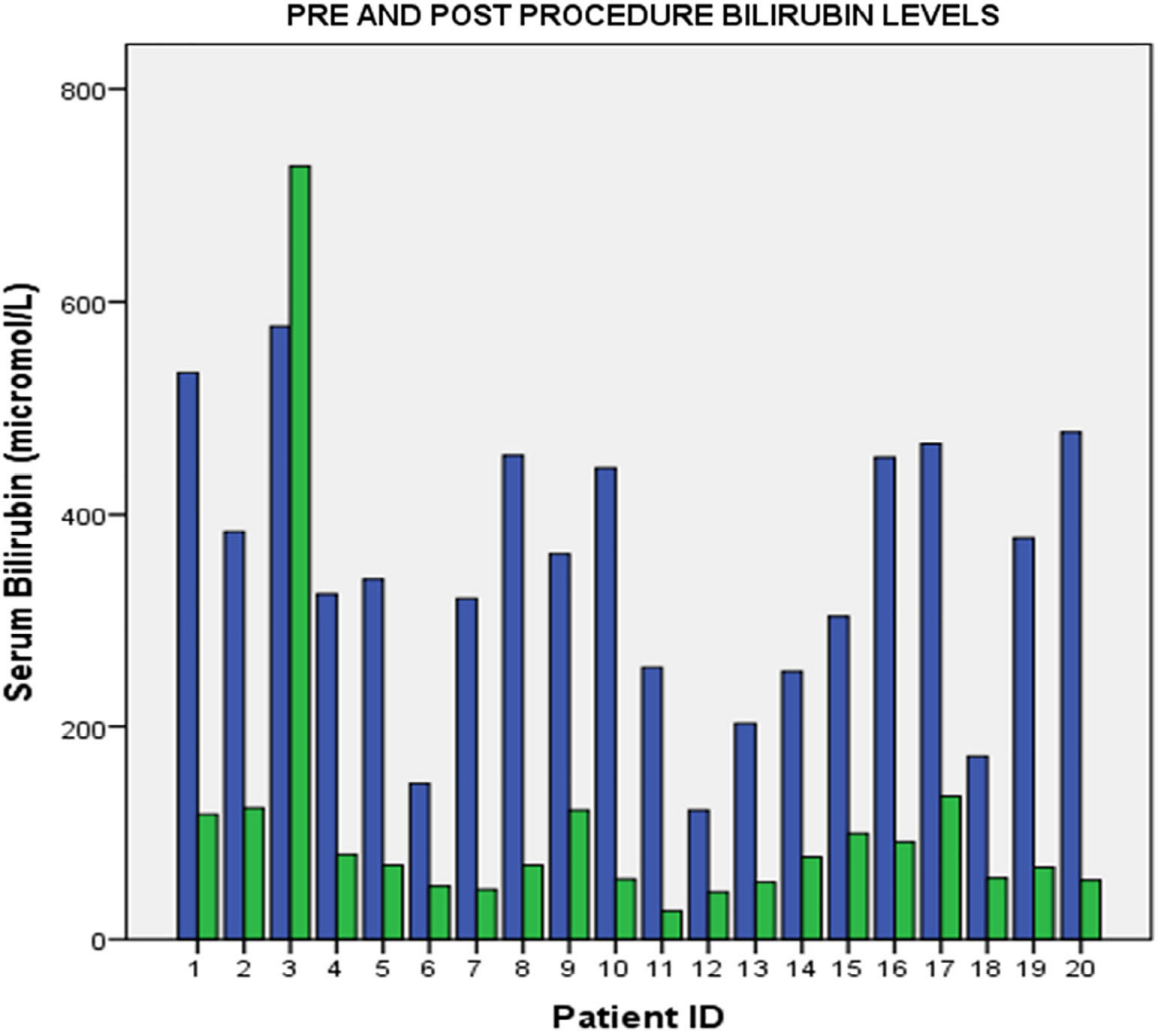
Pre‐ and post‐procedure bilirubin levels. 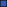, preprocedure bilirubin; 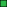, postprocedure bilirubin.

**Figure 3:**
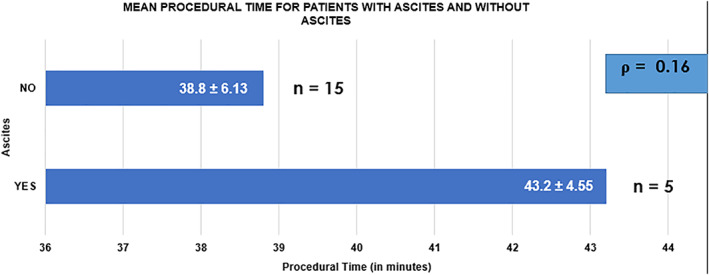
Mean procedural time.

Technical success rate was achieved in all patients. The average procedural time was 39.9 ± 1.3 min. Mean preprocedural bilirubin levels were 348.6 ± 28.8, which decreased to 108.94 ± 37.1 μmol/L at 2 weeks postprocedure. Pre‐ and post‐procedure bilirubin levels are summarized in Figure 2. The clinical success rate was 95% (19/20), with one patient requiring PTBD. Five (25%) patients had ascites, necessitating abdominal paracentesis prior to procedure. All patients with ascites achieved technical and clinical success, albeit with a relatively longer procedural time in comparison to the cohort that did not have ascites (43.2 *vs* 38.8 min, *P* = 0.16). Mean procedural time for both groups are depicted in Figure 3. The average length of stay of the patients was 2.6 ± 2.41 days. There were no complications reported in our study. We conducted a phone call interview for patients 2 months after they underwent their procedure. Three of them were not contactable. All the remaining patients, however, did not have any symptoms to suggest recurrent biliary obstruction. The overall outcome of this procedure is summarized in Table 3.

**Table 3:** Overall outcome of the endoscopic ultrasound‐guided hepaticogastrostomy procedure

Outcome (*n* = 20)	Value
Technical success, *n* (%)	20 (100%)
Clinical success, *n* (%)	19 (95%)
Reintervention rate, *n* (%)	1 (5%)
Stent related complication rate, *n* (%)	0 (0%)
Average length of stay (days)	2.6 ± 2.41

### *Complications*

The stent‐related complications that have been previously reported include bile leak, bleeding, cholangitis, sepsis, and peritonitis. However, there were no complications reported in our study.

## Discussion

This is the first study that evaluated the outcome of the EUS‐BD procedure using the BPD Hanaro PCMS dedicated for EUS‐HGS in patients with malignant biliary obstruction. EUS‐BD is the preferred alternative at our center, largely based on patients preference and it being the more readily available option in comparison to PTBD. Majority of our patients had a failed ERCP due to inaccessible papillae as a result of duodenal stenosis. EUS‐BD has previously demonstrated a superior technical success rate in patients with duodenal stenosis.^16^ We achieved a 100% technical success rate and 95% clinical success rate, which is similar to the previously reported technical success rate of 65–100% and clinical success rate of 87–100% by Takeshi Ogura *et al*.

The limitations of the EUS‐HGS procedure as highlighted by Itoi *et al*. include risk of mediastinitis with the transesophageal approach, complexity of undertaking this procedure in a cirrhotic patient, and the risk of injuring the portal vein.^17^ To curtail the risk of mediastinitis or pneumomediastinum associated with a segment 2 (B2) puncture, we only attempted biliary access via segment 3. We had one patient with underlying liver cirrhosis secondary to hepatitis C who underwent a successful procedure. The technical challenge in a cirrhotic patient would be the underlying liver fibrosis that would offer resistance as the needle traverses the liver parenchyma to access the intrahepatic ducts. However, being a solitary case of a patient with cirrhosis makes it insufficient to generalize its outcome. Other factors that may contribute to the technical failure of this procedure is the displacement between the puncture site of the gastric wall and intrahepatic bile duct.^17^ Worth noting is the presence of ascites that would compound this problem as it would further distance the nonapposed gastric wall and left liver lobe. In the past, massive ascites and underlying gastric cancer have been viewed as a contraindication for the EUS‐HGS procedure as the former would hamper fistula formation, and the latter would leave a reduced gastric volume to permit this procedure.^13^, ^18^ In our study, five patients had severe ascites that required abdominal paracentesis prior to EUS‐HGS; however, all patients underwent a successful procedure. We used the longer stent option (10 cm) for all patients with concomitant ascites due to the risk of stent migration with reaccumulation of ascites.^19^

The only exception to clinical success in our study occurred in a patient who had a history of laparatomy + Roux‐en Y‐gastrojejunostomy (RYGJ) at a different center for metastatic sigmoid adenocarcinoma. She presented to us 4 months after her surgery with obstructive jaundice, and CT imaging revealed metastatic portal lymph node enlargement as the underlying cause. An ERCP attempt for palliative stenting failed in view of the long Roux limb, which is not unexpected after a surgery of this nature. ERCP in a Roux‐en Y anatomy has always been technically challenging for the endoscopist, with a reported success rate of 33% in reaching the papilla.^20^, ^21^, ^22^ Balloon enteroscopy‐assisted endoscopic retrograde cholangiography (BAERC) has been recognized as an alternative tool for performing ERCP in patients with RYGJ anatomy; however, even in instances where the afferent limb can be successfully negotiated to reach the second part of the duodenum, cannulation of native papillae is often a formidable task due to the forward optics and working channel of the enteroscope, as well as the lack of an elevator.^22^ We decided to proceed with an EUS‐HGS, which was a technical success, but the persistently elevated bilirubin levels were an ominous sign that we had failed to achieve biliary decompression. A PTBD was attempted, upon which her liver function test improved, and on Day 3, she underwent internalization of the drainage catheter. The patient was discharged on Day 5 post‐procedure. There were no stent‐related complications in our study, which is inconsistent with previously reported overall adverse event rate for EUS‐HGS, which was 23%.^11^ Nevertheless, in view of our relatively small sample size, it would be difficult to extrapolate these results. (Previous EUS‐HGS studies with associated complication rates are summarized in Table 4).

**Table 4:** Studies related to endoscopic ultrasound‐guided hepaticogastrostomy

Author	Year	*n*	Type of stent	Procedural time, median (range) min	Technical success (%)	Clinical success (%)	Complication rate (%)
Cho *et al*.^23^	2017	21	PCSEMS	18 (11–45)	21 (100)	18 (85.7)	4 (19) Pneumoperitoneum (2) Bleeding (1) Abdominal pain (1)
Minaga *et al*.^24^	2017	30	PS (9) FCSEMS (20)	39.5 (21–68)	29 (97)	22 (76)	3 (10) Bile peritonitis (3)
Sportes *et al*.^25^	2017	31	FCSEMS	NA	31 (100)	25 (81)	5 (16) Severe sepsis (2) Bile leak (2) Bleeding and death (1)
Amano *et al*.^26^	2017	9	PCSEMS	14 (11–18)	9 (100)	9 (100)	1 (11) Abdominal pain
Moryoussef *et al*.^27^	2017	18	FCSEMS	NA	17 (94)	13 (76)	1 (6) Bleeding and death
Honjo *et al*.^28^	2018	49	PCSEMS	NA	49 (100)	‐	11 (22) Abdominal pain (6) Bleeding (5)
Miyano *et al*.^29^	2018	41	PCSEMS	NA	41 (100)	41 (100)	6 (15) Bile peritonitis (4) Cholangitis (1) Stent migration (1)
Okuno *et al*.^30^	2018	20	FCSEMS	34 (16–98)	20 (100)	19 (95)	3 (15) Cholangitis (3)
Current study	2020	20	PCSEMS	40 (27–50)	20 (100)	19 (95)	0

FCSEMS = fully covered self‐expandable metal stent; NA = not applicable; PCMS = partially covered self‐expandable metal stent; PS = plastic stent.

## Conclusion

EUS‐HGS is emerging as an effective and safe alternative in endoscopic biliary decompression following failed ERCP. The hybrid nature and antimigratory properties of the PCMS makes it a valuable option in patients undergoing EUS‐HGS. Despite the failure to achieve clinical success in our patient with surgically altered anatomy, previous published data have demonstrated its applicability in that cohort of patients. It may be difficult to generalize the outcome of our study due to a relatively small sample size.

## Declaration of conflict of interest

None.
